# Effects of Congenital Ocular Toxoplasmosis on Peripheral Retinal Vascular Development in Premature Infants at Low Risk for Retinopathy of Prematurity

**DOI:** 10.4274/tjo.galenos.2019.74484

**Published:** 2019-09-03

**Authors:** Murat Hasanreisoğlu, Şengül Özdek, Gökçen Deniz Gülpınar İkiz, Zeynep Aktaş, Tuba Atalay

**Affiliations:** 1 Gazi University Faculty of Medicine, Department of Ophthalmology, Ankara, Turkey; 2 University of Health Sciences, Van Training and Research Hospital, Ophthalmology Clinic, Van, Turkey

**Keywords:** Retinopathy of prematurity, ocular toxoplasmosis, congenital toxoplasmosis, pars plana vitrectomy, toxoplasma gondii

## Abstract

Congenital toxoplasmosis and retinopathy of prematurity (ROP) are two devastating clinical entities of the newborn. There is little information in the literature about the interaction between congenital infections and retinal vascular development at the fetal stage, and none regarding the relationship between ROP and congenital toxoplasmosis. In this report, we present two premature newborns diagnosed with congenital toxoplasmosis with ocular involvement, accompanied by ROP with interrupted retinal vascularization, peripheral avascular regions, and retinal detachment. The aim of this paper is to emphasize the possibility of ROP and congenital toxoplasmosis coexistence wherein one condition may mask the other and make it difficult to distinguish the cause of retinal detachment. Timely management with medical and surgical treatment of congenital toxoplasmosis and ROP could save eyes and vision in those cases.

## Introduction

*Toxoplasma gondii *is an opportunistic parasite that can cause acquired or congenital infections.^1^ Congenital infection occurs via vertical transmission from the mother and may result in fetal death, severe congenital malformation, fetal developmental retardation, and the infection of neural tissues. The best described clinical presentation of ocular toxoplasmosis is focal necrotizing chorioretinitis, ultimately resulting in characteristic atrophic scars.^1,2^ Retinopathy of prematurity (ROP) is a vasoproliferative, multifactorial disorder of the retina that occurs principally in newborn preterm infants and is strongly related to low gestational age (GA), low birth weight (BW), and supplemental oxygen therapy.^3,4^

It is well known that serious congenital infections such as chorioamnionitis, placental infections, and sepsis increase the risk for ROP in susceptible premature infants.^4^ When the constitutional features and risk factors are taken into consideration, ROP could accompany congenital toxoplasmosis as well, which is the focus of this paper. Here, we present two newborns who were diagnosed and referred to our ophthalmology clinic with congenital toxoplasmosis with ocular involvement, but also had accompanying ROP with interrupted retinal vascularization, peripheral avascular regions, and tractional retinal detachment.

## Case Report

A premature newborn baby girl (Case 1) and baby boy (Case 2) were referred to our ophthalmology/uveitis clinic from the neonatology units for the confirmation of congenital ocular toxoplasmosis. Prenatally, hydrocephaly had been detected in both newborns via ultrasonography. *Toxoplasma* IgG and IgM antibodies were also detected in both mothers during pregnancy. Postnatally, the newborns were evaluated for congenital toxoplasmosis. Infectious markers in blood samples confirmed the diagnosis of congenital toxoplasmosis. Both infants were monitored in neonatal intensive care units, and systemic antiparasitic (pyrimethamine 2 mg/kg, sulfadiazine 100 mg/kg, and leucovorin 10 mg) and anti-inflammatory (prednisone 2 mg/kg) therapy was initiated. Systemic features are given in detail in Table 1. Detailed ophthalmic features of the two cases are given in Table 2.

**Figure 1:**
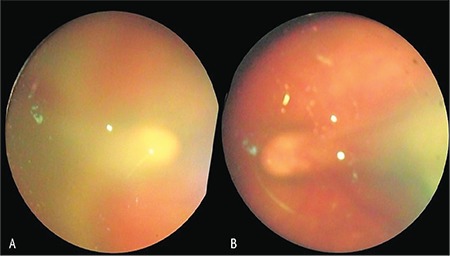
Case 1, fundus appearance at initial visit in the A) right eye and B) left eye; focus of active retinitis at posterior pole inside the vascular arcades starting from optic disc extending temporally, resulting in traction and straightening of the vascular arcades and causing a comet-like appearance of the posterior pole. The view was hazy due to vitritis

**Figure 2:**
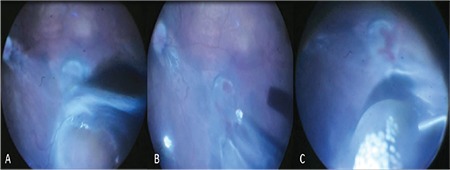
Case 1, intraoperative fundus images of the right eye: A) Peripheral retinal avascular regions and the thick fibrotic membrane at the temporal retina and chorioretinitis lesion causing tractional retinal detachment and narrowing of the angle between arcuate vessels; B) Intraoperative fundus view after peeling off all the fibrotic membranes; C) Endolaser application for peripheral avascular retina

Case 1: She was born at a GA of 34 weeks at a BW of 2230 g, and was post-gestational 40 weeks of age at the time of ophthalmologic consultation. Ophthalmological examination revealed a normal anterior segment in the right eye, with adequate pupil dilation. The left eye was microphthalmic in appearance. Dilated fundus examination of the right eye revealed a circular focus of active retinitis in the macular area with a fibrotic membrane starting from the retinitis area extending temporally to the peripheral retina, resulting in retinal traction and straightening of the vascular arcuates, causing a comet-like appearance of the optic disc. The view was hazy because of +2/+3 vitritis (Figure 1a). Examination of the left fundus revealed a similar lesion temporal to the optic disc accompanied by a foveal fold and traction in the arcuates towards the temporal lesion (Figure 1b). The left eye also had +2 vitritis. Peripheral vascular details were indistinct due to the intense inflammation and retinitis. At this stage, close observation with systemic medical treatment of CT was recommended. The vitritis had subsided by 1-month follow-up, enabling the detection of significant peripheral avascular regions in the temporal retina of the right eye and the fibrovascular membranes overlying these areas. Retinal traction towards the temporal periphery was also noted. The left eye already had seclusio pupilla and lens opacification at this stage. With these findings, the diagnosis was revised as ROP in addition to toxoplasma chorioretinitis. Since the left eye was pre-phthisic, a lens-sparing vitrectomy was planned for the right eye only. Intraoperatively, tractions were released by peeling the fibrotic membranes extending from the macular chorioretinitis scar to the temporal periphery. Laser photocoagulation was applied to the peripheral avascular areas extending to zone 1 in the temporal quadrant (Figure 2a-c) and fluid/air exchange was done at the end of surgery. ROP was graded as stage 2 at the ridge and the tractional membranes were considered to be of inflammatory origin. Postoperatively, the retina was attached, without any macular tractions, with a pigmented scar corresponding to the old chorioretinitis lesion. During the follow-up of 18 months, fundus findings were stable with attached retina. Intraocular pressure was 17 mmHg. She was able to fixate and follow objects with that eye. However, the fellow eye became totally phthisic.

**Figure 3:**
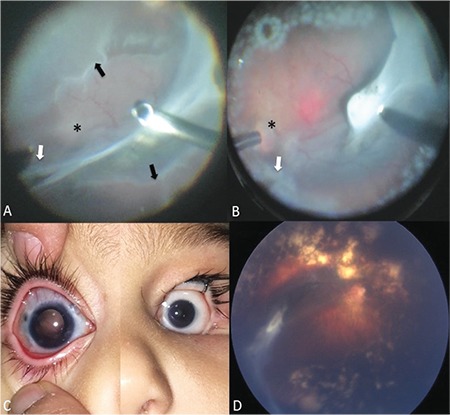
Case 2: A) Combined rhegmatogenous and tractional retinal detachment in the right eye. Note the fibrotic membrane extending from nasal to temporal periphery leading to a large triangular retinal break (white arrow) superior to the optic nerve head (black star), which is barely visible due to the nasal retina pulled over it, and avascular retina in zone 1 with stage 2 retinopathy of prematurity (black arrow); B) Peroperative fundus image after peeling the fibrotic membranes around the large, triangular retinal break (white arrow) and application of endolaser photocoagulation to avascular retina and around the breaks; C) Postoperative image showing the buphthalmic appearance of the right eye and microphthalmic appearance of the left eye; D) Postoperative fundus image of the right eye with attached retina and fibrotic membrane remnants in the temporal area and laser scars visible throughout the periphery

Case 2: He was born at a GA of 32 weeks and BW of 1590 g. He was post-gestational 37 weeks old at time of ophthalmologic consultation. Ophthalmological examination revealed bilateral cataracts and seclusio pupilla. We could detect buphthalmos with Haab’s striae in the right eye (corneal diameters: 12x12 mm, axial length: 25 mm) and microphthalmus (corneal diameters: 7x8 mm, axial length: 13 mm) in the left eye. Right eye fundus examination was unable to provide clues regarding retinal detachment due to cataract and posterior synechia. Ultrasound examination revealed preretinal membranes and freely mobile retinal detachment resembling rhegmatogenous retinal detachment. Lensectomy and vitrectomy were performed on the right eye. During surgery, complete bullous retinal detachment was observed, as well as a thick, tight fibrotic membrane extending from the nasal to temporal retina passing superior to optic nerve head (ONH) and over the macular pigmented chorioretinitis scar, ending in a very thick and strong fibrotic adhesion in the temporal equator. The nasal retina was pulled towards the temporal scar tissue over the ONH. There was a large, triangular retinal break at the nasal end of the membrane superior to the ONH, and the peripheral retina was avascular in zone 1 with a stage 2 ridge at the junction (Figure 3a). The fibrotic tractional membranes were removed meticulously around the break to eliminate all tractions. The posterior hyaloid could be detached from the posterior pole to the ridge area but a small iatrogenic retinal hole formed in the inferior ridge region. Endolaser photocoagulation was applied to the retinal tears and avascular retina (Figure 3b), and 16% C3F8 gas was chosen as a tamponade. At 1-year follow-up, mild buphthalmic appearance with minimal corneal edema persisted, with the addition of minimal scleral staphylomas at the previous sclerotomy sites (Figure 3c). Glaucoma could not be controlled with medical treatment and an Ahmed Glaucoma Valve implantation was performed to prevent further glaucomatous damage. The retina remained attached throughout follow-up (Figure 3d). Vision could not be assessed well because of the mental-motor retardation, but the patient was able to follow light and large objects.

## Discussion

CT is recognized as a major cause of child morbidity and mortality which occurs via vertical transmission from the mother primarily infected during pregnancy. CT may result in fetal developmental retardation and death. A history of hydrocephalus, retinochoroiditis, and calcifications in the central nervous system in the neonatal period should immediately alert a care provider to the possibility of toxoplasmosis.^1,2^ In contrast, ROP is a retinal neovascular disease of premature infants. Despite major advances in management, it continues to be a leading cause of childhood blindness throughout the world. ROP develops when the immature retinal vasculature exhibits a vasoconstrictive response to hyperoxia followed by a vasoproliferative phase that is driven by the surge in endothelial growth factors on the return to normal oxygenation.^3^

Prematurity and CT have different systemic manifestations, yet their ophthalmic manifestations may theoretically overlap. It is also well known that serious congenital infections such as chorioamnionitis, placental infections, and sepsis increase the risk for ROP.^4^ Although not mentioned commonly in the literature, prematurity is a plausible consequence of CT since we know that fetal developmental retardation is a possible effect.^3,4^ Incomplete retinal vascularization in ROP may either be a possible consequence of impaired fetal development resulting in prematurity, or an unexplained clinical presentation of the congenital ocular infection. No matter which etiopathogenetic mechanism is responsible, retinopathy associated with prematurity seems to be a possible comorbidity for these infants. Despite those facts, to the best of our knowledge, our paper is the first to emphasize the possible coexistence of congenital ocular toxoplasmosis (COT) and ROP.

In the modern era of medicine, when a baby is born premature and has obvious risk factors for ROP such as low BW, the diagnosis is straightforward after a good fundus examination.^3^ However, as in the present cases, when the risk factors for ROP are indistinct and the newborn is already diagnosed with other devastating conditions such as COT, it may be challenging to recognize retinal features of ROP among widespread inflammation. In the current report, both babies were not referred for ophthalmological examination for ROP screening, but only for the possibility of COT. This is probably because the babies’ GA and BW were slightly higher than the lower limits for screening. Another important and unique feature of this report is the presentation of the ROP clinical appearance under the inflammatory cover of the COT in these premature babies who had a low risk of ROP. The membranes causing traction in both babies were felt to be primarily inflammatory rather than ROP-related in origin since they were almost avascular, not originating from the ridge areas, and more prominent around the chorioretinitis scar. On the other hand, there were significant avascular zones which could not be simply explained by the child’s BW or GA. That is why the stage of ROP was evaluated as not more than stage 2 in both eyes. It seemed that the inflammation caused by COT promoted progression of ROP in bigger, low-risk babies in whom ROP is not expected and screened routinely.

The classic medical treatment for COT includes a combination of pyrimethamine and sulphonamides.^5^ It is especially important to halt parasite replication in the eye to prevent irreversible damage to the retina and optic nerve that can lead to permanent blindness.^5^ However, for cases with widespread infection in the eye, intercepting the replication cycle may not be sufficient to save the eye and vision due to the severe inflammatory response, even if systemic steroid therapy is also administered. In the presence or absence of advanced ROP, the presence of tractional membranes and retinal detachments should be regarded as surgical indications in the management of COT. In the present report, both cases received systemic antibiotic and steroid treatment, but surgical treatment was also needed to prevent further loss of vision and possible loss of the globe. ROP and CT may mask each other and it may be difficult to distinguish the cause of retinal detachment in such eyes. It is well known that tractional retinal detachment may be caused by advanced ROP (stage 4/5) and this may be confused with the tractional retinal detachment caused by COT.^6^ Fibrotic membrane formation and tractional retinal detachments during the acute stage of COT are rarely mentioned in the literature. This is consistent with the fact that there is hardly any suggested surgical treatment option for COT during infancy in the literature. The current report is not only unique in demonstrating vitrectomy as an effective treatment modality in COT in the presence of inflammatory fibrotic membranes leading to retinal detachment, but also in enabling the endolaser photocoagulation to treat avascular zones that were attributed to ROP.

In summary, these two cases emphasize the fact that two different retinal diseases, COT and ROP, may be intertwined in preterm infants and present with atypical manifestations. Therefore, in addition to history and routine fundus examination, peripheral retinal vascularization should also be carefully evaluated in bigger babies with COT.

## Figures and Tables

**Table 1:**
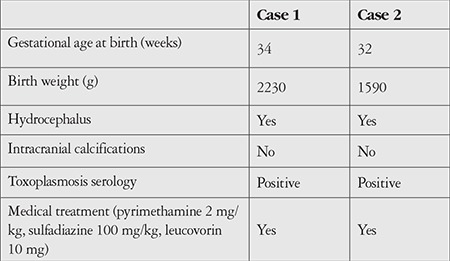
Clinical features and treatment of two patients with retinopathy of prematurity and congenital toxoplasmosis

**Table 2:**
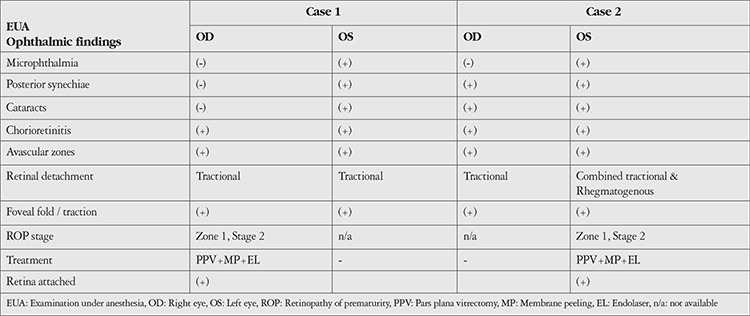
Main ocular features, treatments, and outcomes in the two cases
